# Problems and Their Solutions in Genetic Counseling Education in Japan

**DOI:** 10.3389/fpubh.2014.00100

**Published:** 2014-07-29

**Authors:** Hidetsugu Kohzaki

**Affiliations:** ^1^Department of Cell Biology, Institute for Virus Research, Kyoto University, Kyoto, Japan; ^2^Department of Molecular Medicine, Graduate School of Medicine, Osaka University, Suita, Japan; ^3^Faculty of Allied Health Science, Yamato University, Suita, Japan

**Keywords:** genetic counselor, genetic literacy, ICT skill, employment, education

## Abstract

With the expansion of novel chromosome testing, a career as a certified genetic counselor has been gathering a lot of attention. However, few people certified as a genetic counselor after completing postgraduate courses are able to find employment as a genetic counselor, and their salaries are quite low. It is also questionable whether or not such newly graduated genetic counselors, who have limited life experience and knowledge, can fully understand family issues and properly perform counseling sessions. To address these issues, a wide range of education and training may be necessary. In this study, we examined current problems in genetic counseling education in Japan, and proposed effective measures to address these problems. Toward creating a new society, we are currently establishing a national qualification system and cultivating qualified professionals capable of providing patients with accurate information on chromosome and genetic testing. In addition, these professionals could encourage younger generations to have an interest in genetic counseling. I also hope that these professionals will work not only in Japan but all over the world.

## Introduction

Early on, in Europe and the United States, genetics education was conducted in terms of human genetic diseases. According to WHO guidelines, genetics education from elementary school is recommended. In Japan, students have mainly studied Mendel’s law (1–3).

Many years have passed since the completion of the Human Genome Project. Based on the information obtained from the project, personalized medical practice, specific gene tests, genetic tests, and pharmacogenomics have developed in Japan (4–11). A number of authors have described genetic counseling graduate programs in other countries that educate professionals to assist individuals in making informed decisions regarding their genetic risks [e.g., Ref. (12, 13)].

In Japan, promotion of genetic counseling (previously called genetic consultation) as defined by the American Society of Human Genetics (ASHG) began in the 1970s. At that time, genetic counselors were mostly basic researchers, medical researchers, and physicians (especially in the fields of pediatrics and internal medicine) (14). However, genomic medicine has developed and gene diagnosis has been practically applied since the 1980s. These services markedly changed how hereditary diseases were understood, because information and testing options could be provided to patients and families about not only congenital diseases and diseases inherited in a Mendelian fashion but also cancers and lifestyle-related diseases. These developments also placed increased demands on the knowledge and skill base necessary for effectively providing genetic counseling.

In response to these developments, the Japanese Society of Human Genetics (JSHG) (15) and the Japan Society for Genetic Counseling (JSGC) (16) established guidelines for genetic counselors in Japan. Genetic counselors are required not only to provide genetic medical information but also to assist patients in managing the psychosocial ramifications of their genetic risks and/or genetic condition. Knowledge of genetic information and psychological management techniques are necessary for effective service provision. In addition, the nature of genetic counseling services and the issues experienced by patients and providers pose a number of ethical challenges. Given the necessity for extensive scientific (genetic) knowledge, psychological intervention skills, and ethical reasoning ability, specialized genetic counselors, independent from physicians, are essential.

In Japan, various practical guidelines have been established (4–11). Of particular note, in Japan, genetic counseling is performed according to established guidelines, which prohibit genetic counselors from providing other services. Genetic counselors generally seek to promote the well-being of patients and their families in cooperation with treatment and management recommendations from physicians. Genetic counseling is concerned with very personal topics affecting patients and their families. Table 1 contains a list of components that comprise a typical genetic counseling session in Japan. In addition, the training program of genetic counselors in Japan is noted in the websites of Genetic counselors (17) and the JSHG (15).

**Table 1:** Components and methods of genetic counseling in Japan*.

1. Introduction of interview – self-introduction and confirmation of session goals
2. Case preparation – prior preparation corresponding to individual cases, includes medical/genetic and psychosocial considerations
3. Family history taking – inquiring about familial and past medical histories and confirmation of the diagnosis
4. Risk assessment – corresponding to individual case
5. Provision of information regarding inheritance – information about the condition and probability of inheritance
6. Explanation of condition – prognosis and treatment/management
7. Discussion of options – genetic testing options and options following receipt of test results
8. Assessment of psychosocial and familial issues – for patient and family members
9. Facilitation of patient decision-making
10. Referral – as appropriate, to psychological professionals or social workers
11. Provision of information concerning resources – childcare, social services, patient organizations, etc.
12. Review and follow-up

*Adapted from descriptions of genetic counseling in the U.S. (12, 13).

## Genetic Counseling in Japan

In order to become a genetic counselor in Japan, it is necessary to graduate from a 4-year university course, have operational experience as a professional of medical welfare of 5 years or longer, and take courses on human genetics, counseling, psychology, bioethics, statistics, physical education, role playing, and other subjects in a graduate school in principle. After completing a thesis and graduating from the school, it is possible to take a certification examination to become a genetic counselor, and those who pass become qualified.

Genetic counselors work in genetic service settings, such as universities, pediatric clinics, and general hospitals (4–11). They provide genetic services through a collaborative approach with attending physicians and clinical geneticists. Clinical geneticists are responsible for diagnosis, treatment, and genetic counseling, whereas genetic counselors listen carefully to clients (e.g., patients who have worries about their genes or blood relatives), always respect patients and their family members, provide genetic and related information, and plainly explain the medical, psychological, and familial effects of genetic diseases. Genetic counselors also help clients self-determine in order for them to clearly understand the provided information and explanation, and adapt well to their situation. In other words, the role of genetic counselors is to think with clients and help them overcome their worries. Although genetic counselors sometimes recommend chromosome and gene testing or prenatal testing for clients, the opinions of clients and their family members are respected. When there is a disagreement between a husband and wife about undergoing such testing, genetic counselors help them self-determine after careful deliberation (4–11).

## Challenges in Genetic Counseling in Japan

Aging of the Japanese population is rapidly progressing; conditions such as dementia and other medical concerns of elderly people are increasing demands for nursing care. When such people become patients, it is extremely difficult to explain chromosome and gene analysis, diagnosis, and treatment in ways they can comprehend. Moreover, patients of any age may become anxious, thus further inhibiting their ability to comprehend complex genetic information. Therefore, it is imperative for paramedics (including medical technologists, nurses, care workers, and social workers) that play roles in team medicine to be knowledgeable about genetic testing, genetic conditions, and genetics, and also have the skills for explaining this information to patients. In Japan, it is not sufficient just for physicians to provide accurate and straightforward information to patients in order for them to make a decision. In addition to physicians, support by paramedics may be important considering the length of time they spend with patients. The medical fee structure was recently revised in Japan, and genetic counseling and chromosome and gene testing became widely covered by health care insurance. For example, the fee for genetic counseling is 5,000 yen for use of services once per month (about 60 U.S. dollars). Thus, these services are likely affordable for many patients.

## Need for Specialized Genetic Counseling Services

As described above, Japanese were previously ashamed of having a handicapped child and they tended to be pessimistic about the child’s future. Moreover, many people who have no one with a congenital abnormality in their lives regard congenital abnormalities as unusual and not of concern to them. Most Japanese, even paramedics, may not know that congenital abnormalities are the cause of 1/3 of neonatal deaths (18). In point of fact, gene abnormalities are quite common, which is not known by most people (including the general public and health care workers) other than clinical genetics specialists. Several newborns in 100 are born with congenital abnormalities (1 in 20 when congenital abnormalities are not diagnosable immediately after birth, such as intellectual disability, and hereditary diseases that develop over time are included) (5, 18–23). These data suggest that about 10 genetic changes associated with autosomal recessive hereditary diseases are present in any person, and chromosomal aberrations are detected in 10–20% of male and female gametes in chromosomal testing. Since these gametes with chromosomal aberrations are also involved in fertilization, it is considered that many fertilized ova have chromosomal aberrations in an early stage (the frequency is estimated to be higher than 50% in some reports). The general incidence of newborns with chromosomal aberrations is about 1%, showing that miscarriage occurs in most embryos with chromosomal aberrations (only about 15% of miscarriages are clinically confirmed, but many occur in the early stage of pregnancy before the mother knows she is pregnant) (18). It is important to understand that congenital abnormalities and genetic changes may occur in any individual (19), and thus genetics education is necessary.

When chromosomal aberration additionally occurs in translocation carriers, it is difficult to determine the theoretical probability. Some patients seek genetic counseling about chromosomal aberration due to influences of drugs and radiation. However, it is impossible to identify what chromosomal aberration is induced by which environmental factor, excluding specific chromosomal aberrations, because fertilized ova with chromosomal aberrations are frequently produced in any individual (18, 19).

When an abnormality is detected by genetic testing, pregnancy termination is considered; however, no legal provision currently exists concerning fetuses before 22 weeks of pregnancy in Japan, and therapeutic abortion is performed based on lawful reasoning in accordance with the Maternal Protection Act. The decision is between doctors and their patients (24). In genetic counseling, information concerning prenatal diagnosis, such as methods, costs, risks of tests, and medical institutions performing the diagnosis, is provided to patients for their independent decision-making. In a serious situation, such as a decision regarding therapeutic abortion, counselors support the couple to make a decision after fully discussing it with them and after consulting with the attending gynecologists (4–11, 18, 19). A growing number of patients are concerned about their genetic risks (24). Reportedly, nearly 70% of people develop diseases involving genetic factors in the course of their lifetime (e.g., lifestyle-related conditions including hypertension, diabetes, cancer, and autoimmune diseases). Upon the completion of human genome sequencing, studies to elucidate multifactorial genetic diseases (diseases associated with several genetic abnormalities and environmental factors) have proliferated (12–14).

Hereditary diseases that the general public has never heard of now create anxiety, and concerned individuals visit many hospitals. Hospitals in Japan have increasingly opened departments specializing in hereditary diseases, such as gene therapy and hereditary disease treatment. Several departments specializing in hereditary diseases have developed websites offering information about facilities providing genetic counseling and containing the latest genetic medical information. For example, “Iden Net” (25) is mainly operated by the Clinical Genetics Unit, Kyoto University Hospital, Kyoto, Japan. Facilities performing genetic consultation and testing are listed, and various guidelines and explanatory documents for patients are presented. Another example is “Genetopia” (26) operated by the Division of Clinical and Molecular Genetics, Shinshu University Hospital, Nagano, Japan. Various kinds of information concerning inheritance are presented, and patients can access contents for medical workers.

## Discussion

### Recommendations for “genetic literacy” education

Recently, the position of genetic nurse has been established in the UK, and training for this position has begun in Japan. According to WHO guidelines, not only physicians but also all health care providers, such as nurses and public health nurses, are advised to have the ability to provide genetic counseling irrespective of their professions. According to WHO, the profession and population ratio of medical geneticists in the advanced countries is about 1:220,000, and about 1:3,700,000 in developing countries and Japan. Therefore, Japan needs to accelerate further training (2, 27).

Genetics is not taught in Japanese high schools (28, 29). In Japan, medical technologists used to perform chromosome and genes analysis on a routine basis. However, as shown in Table 2, their genetic knowledge was insufficient, leading to the establishment of a new profession, as mentioned in the conclusion. The general public is far more genetically illiterate (28, 29).

**Table 2:** **My proposals**.

1. Improvement of English and ICT skills
2. Acquisition of a broad knowledge of medical genetics
3. Recognition of ethics and compliance
4. Increasing hours of training and learning to ensure accurate testing
5. Enhancement of training facilities
6. Improving the quality of teacher training education
7. Reducing the burden of clinical geneticists and obstetricians
8. Contributing to team medicine

### Necessity of “genetic literacy” education throughout the world

Owing to the advice of the ASHG (30) and the European Society of Human Genetics (31) and influence of the WHO guidelines, Japanese societies for chromosome and genes analysis, such as the Japan Society of Human Genetics, have improved. The WHO has also recommended that not only physicians but also all healthcare providers become genetically literate, and that human genetics education be introduced from the early stages of education, such as elementary or junior high school (27, 28).

Chromosome and genes analysis technologies have been advancing rapidly, such as the PCR and genome-wide association study (GWAS) (28). Recently, SEQUENOM, Inc., developed a new prenatal diagnosis, which is almost certainly know whether there are some chromosomal abnormalities such as Down’s syndrome in the fetus by just look at the blood of only 10 weeks pregnant women. In Japan, their due is 200,000 yen (32). We must teach students the recognition of ethics and compliance and recommend renewals of genetic literacy repeatedly.

Given such advancement, human genetics must be studied by healthcare providers engaged in chromosome and genes analysis as well as the general public of not only East Asian countries but also countries throughout the world.

### My proposals

I have provided some proposals about the improvement of the quality of genetic counseling education (Table 2). Since most counselors rely on automatic analysis equipment, the foundations of education are skills and skilled understanding of principles, especially English (33), ICT (which is improving rapidly year by year) (34, 35), basic molecular biology, and clinical genetics. To ensure accurate practical testing, it is necessary to provide hours of training to learn the procedures. Most importantly, it is necessary to improve the quality of teacher training education.

## Conclusion

In Japan, employment of genetic counselors is a critical problem. It has been difficult for genetic counselors to find desired employment. Because they are not required to obtain a national qualification, their position is not well accepted in the medical field. In addition, salaries are quite low for genetic counselors. Therefore, it is necessary to improve their work situation and modifications are required in order to meet the current needs of Japanese society.

In order to address the problems described in Table 2, we aim to create a new society by establishing a national qualification for (which a certain level of fixed income is guaranteed) system for “genome consultants” (or “genome inspectors”) (tentative names). These new professionals, on equal footing with physicians, would provide patients with accurate information on chromosome and genetic testing in cooperation with physicians, medical geneticists, and medical technologists. In addition, they could encourage younger generations to have an interest in genetic counseling. I also hope that these professionals will work not only in Japan but all over the world.

## Conflict of Interest Statement

The author declares that the research was conducted in the absence of any commercial or financial relationships that could be construed as a potential conflict of interest.
